# Impure Blood

**Published:** 1884-03

**Authors:** 


More time will be spent in considering the other great branch of causes, *Impure Blood*, because it is not generally understood what are the more general causes of impure blood, and they ought to be generally known.

The heart has two suits of rooms, one filled with impure blood, going to the lungs to be purified; the other containing the purest blood of the body, which having undergone purification and perfection in the lungs, has been returned to this other side of the heart, to be propelled therefrom to the most distant portions of the human frame, imparting in its progress, renovation, restoration and life. The right side of the heart contains the impure, imperfect blood, while the pure blood is found in the left. But it cannot get from the right side into the left, without passing through an out-house, the Lungs, where the purifying process is carried on; and how?

We have seen that the blood is in the little branches of blood-vessels spread like a vine on the walls of the air-cells, the lungs, distended by air. Now, the blood does not come in actual contact with the air, the membrane of these minute vessels, thinner than the thinnest paper, manufactured only in Heaven, by omnipotent skill for the express purpose, is between the air and the blood. But a most wonderful process goes on here; there is a passage of substances through these membranes, the life of the air, the oxygen, as we say, passes out of the air-cell into the blood in the blood-vessels, and the impurities, the death of the blood passes from the blood-vessel into the air-cell, and in a moment the dead blood is made alive, and the air so pure from without but a moment before, is now deadly. So the death of the blood and the life of the air pass through these membranes, as light passes through glass or as electricity along the wires. Thus the Lungs are the great 'Change of life—the market place where Vitality and Death change their wares, the air being the nobler of the two, for while it takes death from the blood, it gives its own life therefor, the savior of physical humanity.

Let the most careless reader note and feel here, how impossible it is for the blood to be purified unless he breathes abundant pure air. The importance of breathing it constantly is strikingly exhibited in the established fact, that every ounce of blood of the whole body is thus aired every two and a half minutes of our existence. Thus the breathing of a pure air for so short a time as two and a half minutes imparts purification and refreshment to the whole human frame. This explains the instantaneousness with which persons are revived when taken into the air after confinement to a close room or crowded apartment for some time.

Thus it is, that after writing, or reading, or sewing, in one position for a long time, and the whole body feels tired, we get up, stretch the body, draw a full deep breath and walk across the room a few times, there is a feeling of rest and refreshment comes over us which is most agreeable. Why? Because the full breath distends the air-cells, straightens the blood vessels, the blood passes onward, presenting itself as it passes, to the life giving influences of the air in the freshly and fully distended air vessels. What madness it is, what deliberate suicide, to repress these yearnings of our instincts for the life giving agencies which a beneficent Providence has thrown around us with such bounteous profusion: the Pure Air of Heaven!

But how does the blood become thus impure at the right side of the heart, before it goes for renovation to the lungs? There are two sources of impurity. A barrel of the purest water will be sadly defiled, if taken to the attic, and every floor in the house is washed with it, down to the cellar. The blood starts from the lungs pure and clean, it goes through the whole frame, washing out as it goes along, all the particles of our body which have died since the last visit; for we are always dying reader! Particles which have subserved their uses, and having answered the great end of their creation, must be swept away as the cinders from the grate or the ashes from the hearth. Thus the blood, so pure but two and a half minutes before, is now loaded with offal, and is deposited in the heart, the great Clearing House of the body. So this body of ours is swept out, is washed clean every two minutes and a half of our existence. Like a magnificent steam engine requiring the constant attendance of the engineer, who if he does his duty, is all the time cleaning and oiling, so as to keep it in perfect working order, so is our body.

Does not the reader see, then, that not only is the want of full breathing a cause of impure blood, but that if the air he breathes is not pure when first breathed, it can no more unload the blood of its impurities as perfectly as it ought to have been done, than dirty water can wash a garment clean? You, who habitually breathe an impure, that is, confined air, for all confined air is impure, are a moral suicide. Hurry then, from your bed-chamber the instant of rising; hoist the windows of your sitting apartments, fling wide open your doors, divers times daily, even in the coldest weathers, and let out the death, instead of drawing it into your own system, to fester, and corrupt and rot you.

The other great cause of blood impurity at the right side of the heart, is the following:

We eat to live. What we eat is turned into blood, the object of that blood is two-fold. First, to keep us warm. Second, to repair the wastes of the system. Washing these wastes away in the manner we have named, is a matter of secondary importance, as to the blood; it is rather an incidental work. To keep us warm and to repair, these are First Things.

